# Few more hidden variables which would fortify person centred approach of self-regulated learning

**Published:** 2019-11-28

**Authors:** Dinesh Kumar. V

**Affiliations:** 1 Department of Anatomy, Jawaharlal Institute of Postgraduate Medical Education and Research, Puducherry, India

Dear Editor,

With great enthusiasm I read the article by *Vilppu H et al*.^1^ regarding the application of person centred approach in identifying regulation strategy profiles among students and would like to commend the authors for highlighting self-regulated learning. With the increasing vastness of knowledge found in the literature, self-regulated learning patterns play an increasingly crucial role in harnessing the lifelong learning skills of medical graduates. In addition to the points brought out by the authors, I would like to emphasize motivation and epistemological beliefs in determining the persistence demonstrated by the students towards life-long learning.

Firstly, when a student enters the medical school, he/she possess a certain degree of motivation. In addition to the learning strategies, the baseline motivation also gets modulated by self-regulatory ‘epicycles’.^2^ In developing countries like India, students practice a reproduction-directed approach to learning involving rote memorization in schools and when they try to apply the same way for learning voluminous subjects such as anatomy, they tend to get demotivated easily. This is one of the problems we frequently encounter during mentoring sessions. Rather, I could say that the inability to find the appropriate learning strategy tends to affect the level of motivation demonstrated by students towards learning the content and a significant proportion of struggling learners get lost in this vicious cycle.

Some students, after learning lessons from the initial failures, try to figure out that their level of motivation could get boosted up if they would accomplish smaller sub-goals every day.^3^ At the end of an interesting study, *Eckerlein N et al*.^4^ postulated that motivational difficulties i.e. struggle of students to keep the motivational level higher up while studying tend to vary on a daily basis and it could not be considered a person-specific variable. In addition, these motivational difficulties tend to alter the amount interest students have towards learning. For example, when a student starts feeling that the test portion is too difficult, he/she encounters motivational difficulty. If the same persists, he/she might lower the amount of effort invested towards preparing for the exam and potentially, land up in failure.

Secondly, the epistemological beliefs i.e., students’ self-held perceptions about the quantum of human knowledge possessed by him/her, tend to play a significant role in self-regulated learning. When students are given a task during self-directed learning sessions, some students try to master things quickly from the given content and they tend to correlate it with positive self-efficacy. On the other hand, a proportion of students try to look out for the relative certainty of knowledge and do not get satisfied unless they figure out specific meanings or contexts. *Schommer*,^5^ in her experiment, gave a controversial text to two categories of students and found that the outcomes differed according to the epistemological beliefs. According to her, these epistemological beliefs influences students in choosing the cognitive strategies and influences the resultant performance in academic tasks.

Thirdly, as suggested by the authors, it is imperative to diagnose the learning difficulties of students in order to support their learning regulation profiles. Particularly, some students might possess the cognitive pre-requisites for effective self-regulated learning but are still unable to incorporate the strategies into day-day usage.^6^ Owing to this deficiency, they might suffer from knowledge-action gap and in most cases, analysing under motivational regulation lens might offer the required solution.

To conclude, I support the usage of person-centred approach to help medical educators in explaining the micro-dynamics of learning. However, from a practical perspective, if the motivation regulation profiles of the students were not well understood, the person-centred approach would not be holistic. In addition, it is prudent for the medical educators to recognize diversity with respect to epistemological beliefs in self-regulated learning. Identification of individualized shortcomings should be the prime step for person-centred approach, especially while making remediation plans for struggling learners.
